# Cross-sectional area of the femoral vein varies with leg position and distance from the inguinal ligament

**DOI:** 10.1371/journal.pone.0182623

**Published:** 2017-08-14

**Authors:** Dorota Czyzewska, Andrzej Ustymowicz, Radoslaw Kowalewski, Anna Zurada, Jaroslaw Krejza

**Affiliations:** 1 First Department of Radiology, Maria Sklodowska-Curie Memorial Cancer Center, Institute of Oncology, Warsaw, Poland; 2 Department of Radiology, Medical University of Bialystok, Bialystok, Poland; 3 Department of Vascular Surgery and Transplantation, Medical University of Bialystok, Bialystok, Poland; 4 Department of Anatomy, Faculty of Medicine University of Varmia and Mazury in Olsztyn, Olsztyn, Poland; 5 Institute of Innovative Medicine, Advanced Biomedical Image Laboratory, Bialystok, Poland; Szegedi Tudomanyegyetem, HUNGARY

## Abstract

**Purpose:**

The risk of complications associated with femoral venous catheterization could be potentially reduced if the procedure was performed at the location where the cross-sectional area (CSA) of the vessel is the largest. The diameter of the femoral vein depends on leg position as well as the distance from the inguinal ligament. We determined the CSA of the right femoral vein in three different leg positions at two distances from the inguinal ligament.

**Subjects and methods:**

Informed consent was given by 205 healthy volunteers aged 19–39 years, mean: 23±3 years (108 women, 97 men). Ultrasonographic examinations were performed using a linear 14-MHz transducer with CSA measurements in three leg positions: abduction, abduction+external rotation, abduction+external rotation+90° knee flexion/frog-leg position; at levels 20 mm caudally to the inguinal ligament, and 20 mm caudally to the inguinal crease.

**Results:**

We found significant differences in mean values of CSA in three leg positions regardless of the measurement level. The largest mean CSA (114 mm^2^±35 mm^2^) was found at the proximal level in the frog-leg position. There was a significant association of the CSA with sex and height. The CSA in males was greater than in females in all leg positions at the level of 20 mm caudally to the inguinal crease, while 20 mm caudally to the inguinal ligament the CSA was larger in females. The CSA of 25% of the femoral vein was smaller than 45.0 mm^2^ at the proximal level, and 31.5 mm^2^ at the distal level, which refers to diameters of 5.3 mm, and 4.5 mm, respectively.

**Conclusions:**

The cross-sectional area of the femoral vein is the largest in the frog-leg position, and depends on gender.

## Introduction

Central venous catheterization is a common procedure with over 5 million being performed in the United States every year [1]. Among cannulated vessels, femoral vein access is considered to be one of the easiest [2–4]. According to guidelines, the site of catheter insertion into the femoral vein should be located 2 cm caudally to the inguinal ligament and 1 cm medially from the femoral artery [5, 6]. However, in a case of difficulties in identification of the course of the inguinal ligament under layers of superficial tissues using a palpation method, the inguinal crease is commonly chosen as a landmark for the venipuncture location.

Femoral vein catheterization is associated with risk of failures and serious complications including retroperitoneal hematoma, misplacement of the catheter into the peritoneum and perivascular hematoma [1, 7, 8]. The risk of adverse events related to the procedure could be most likely reduced with choosing the puncture site where the cross-sectional area of the vein is the largest, the distance between the skin surface and the vessel is the shortest, but also the distance from the inguinal ligament is the greatest (to avoid retroperitoneal hematomas). Different approaches were proposed to make the femoral vein more accessible, including Valsalva maneuver, reverse Trendelenburg position, abdominal compression or positive end-expiratory pressure (PEEP) [9, 10]. However, there are studies showing that simple change of patient’s lower limb position can significantly increase femoral vein’s size. In a particular patient, the diameter of the femoral vein and its location in relation to the femoral artery can vary with leg rotation and abduction [11–15]. Therefore, changing leg’s position it is a fast and simple method of increasing femoral vein’s size.

We undertook a study to determine the CSA of the right femoral vein in different leg positions at two different distances from the inguinal ligament and the inguinal crease. We also studied the relation between the CSA of the femoral vein and patient’s sex.

## Materials and methods

### Participants

The study was approved by the Ethics Committee of the Medical University of Bialystok and each participant gave informed written consent. Ultrasound (US) examination was performed on 205 healthy volunteers, aged 19–39 years, mean: 23 ± 3 years (108 women and 97 men), who were recruited among the students of the Medical University and the post-secondary School of Health. Exclusion criteria were: lower limb varices, history of leg surgery, trauma, infection, or thromboembolic events. Study participant characteristics are presented in Table 1.

**Table 1:** Demographic data of the studied participants included in the analysis.

	All, n = 205	Female, n = 108 mean ± SD	Male, n = 97 mean ± SD
Age	22.9 ± 3.4	22.0 ± 2.6	23.9 ± 4.0
Height [m]	1.73 ± 0.09	1.68 ± 0.06	1.80 ± 0.07
Weight [kg]	69.5 ± 13.5	61.4 ± 9.4	78.5 ± 11.5
BMI [kg/m^2^]	22.3 ± 3.2	21.8 ± 3.0	24.1 ± 3.0

### Study design

**Fig 1:**
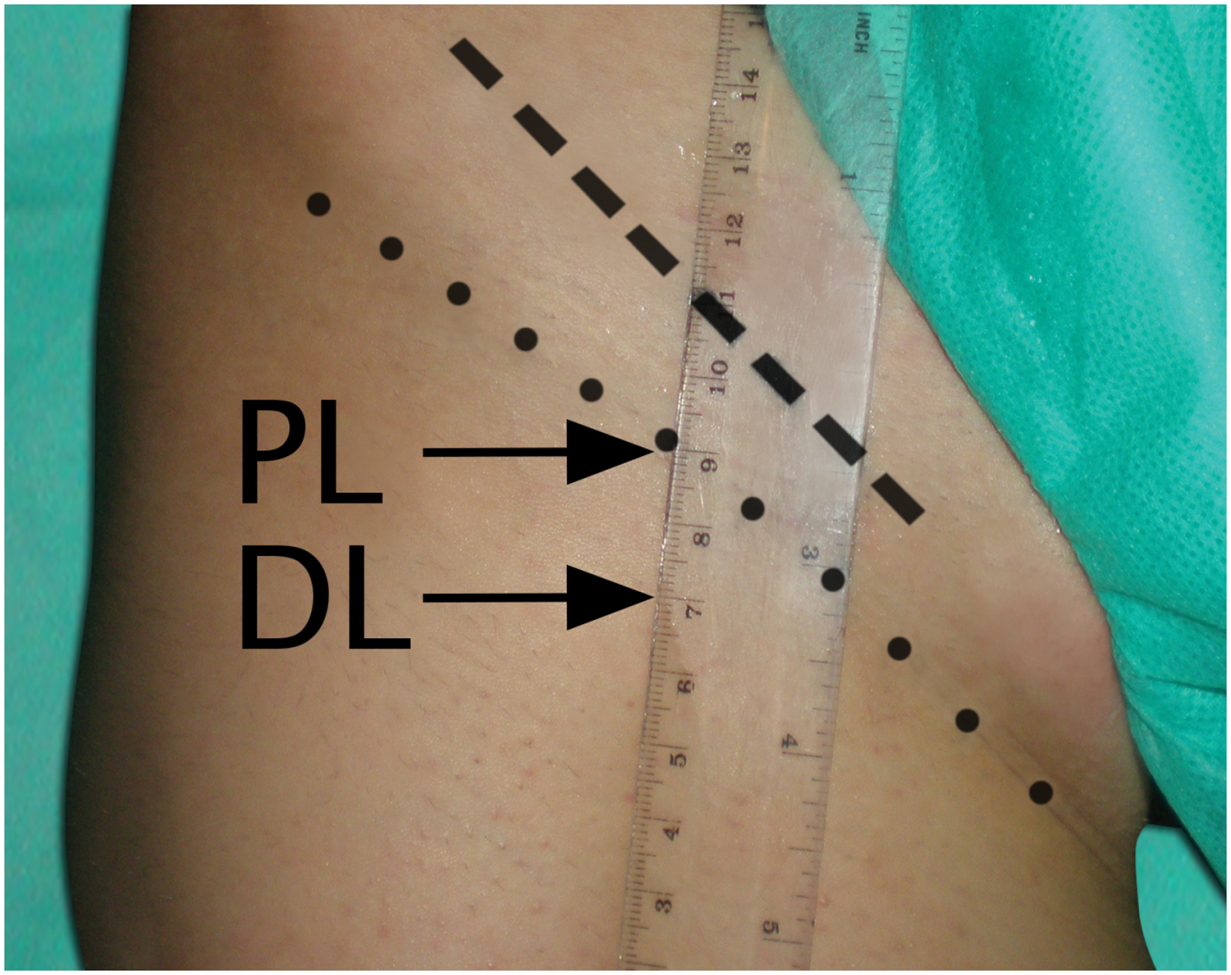
Places of identification of the inguinal ligament and the inguinal crease and levels of measurements of the cross-sectional area of the right femoral vein. striped line—the inguinal ligament, dotted line—the inguinal crease, PL—proximal level, DL—distal level, the ruler represents the path of the femoral artery.

All ultrasound examinations were performed by an experienced radiologist (DC, 5 years of experience) in B-mode technique using a Toshiba-Aplio XG ultrasound machine (Toshiba Medical Systems Corporation, Otawara, Japan), equipped with a linear 14-MHz transducer. The right femoral vein and the inguinal ligament were examined in a supine position. The inguinal ligament was identified along its longitudinal axis and the distance between the inguinal ligament and the inguinal crease was measured along the axis of the femoral artery (Fig 1). Then, the measurements levels were identified as follow:

- proximal level (PL): 20 mm caudally to the inguinal ligament identified along the femoral artery imaged with US (Fig 1)
- distal level (DL): 20 mm caudally to the inguinal crease visually identified and along the femoral artery imaged with US (Fig 1).

**Fig 2:**
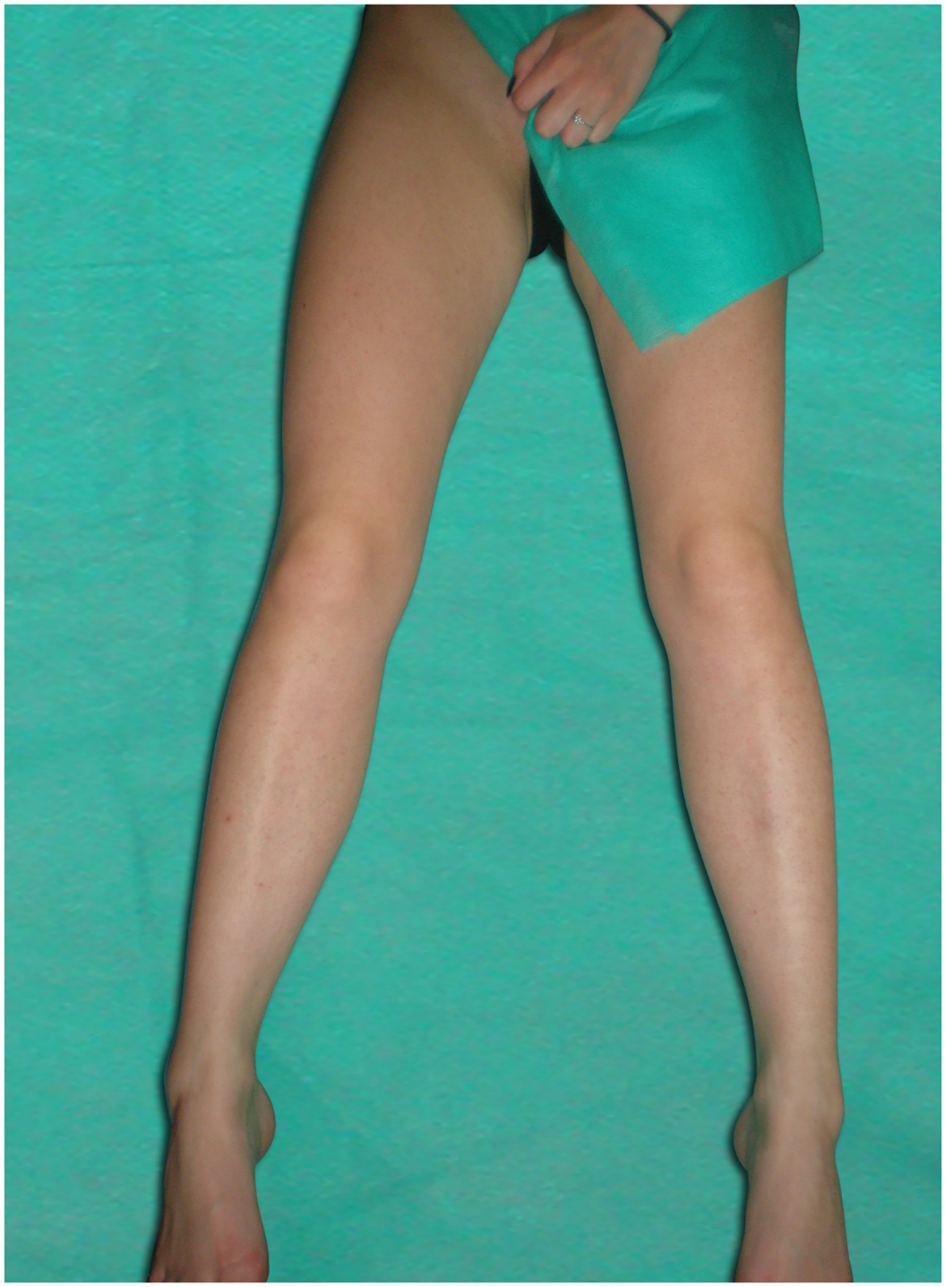
Leg position: Abduction.

**Fig 3:**
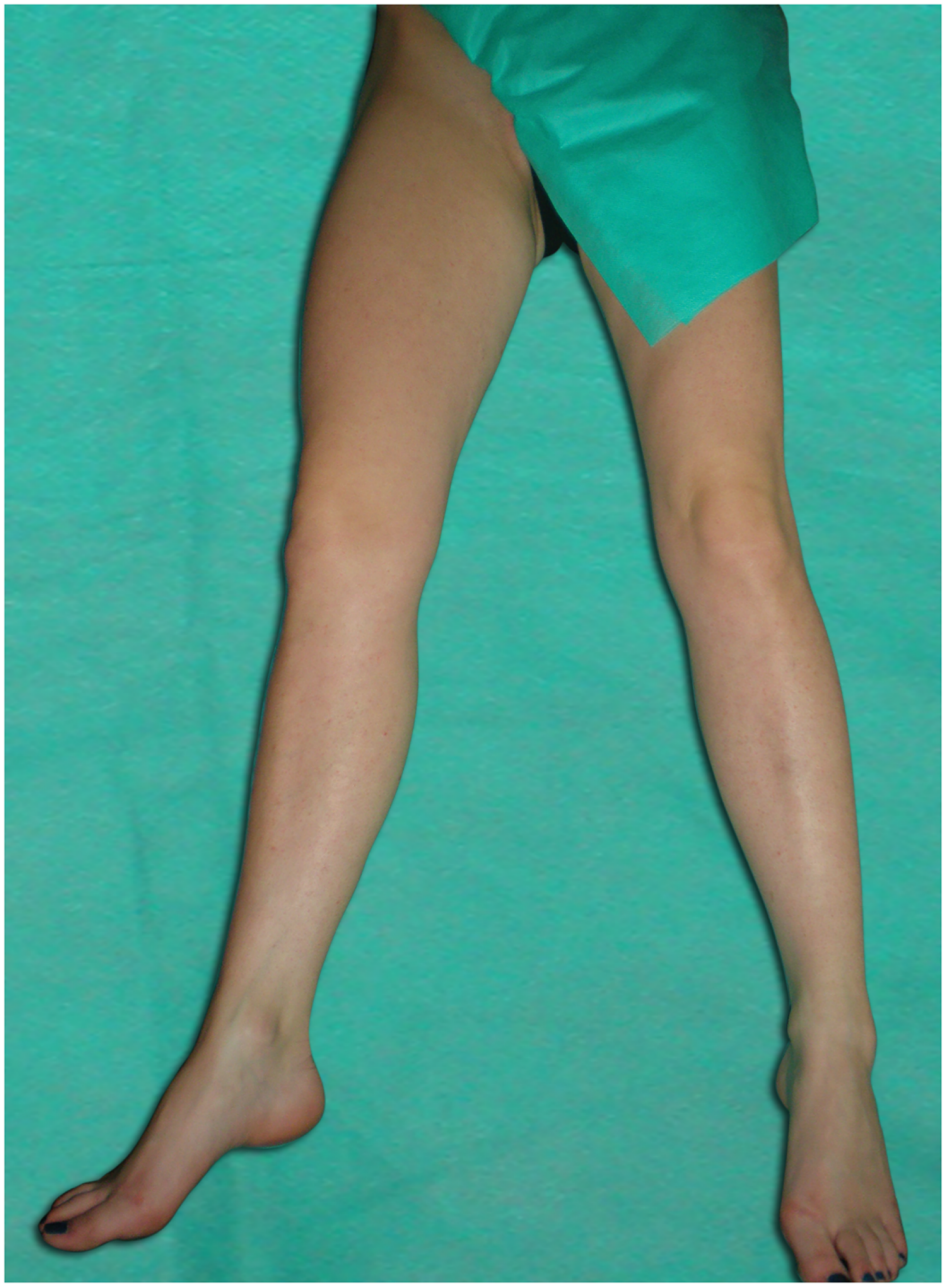
Leg position: Abduction + external rotation.

**Fig 4:**
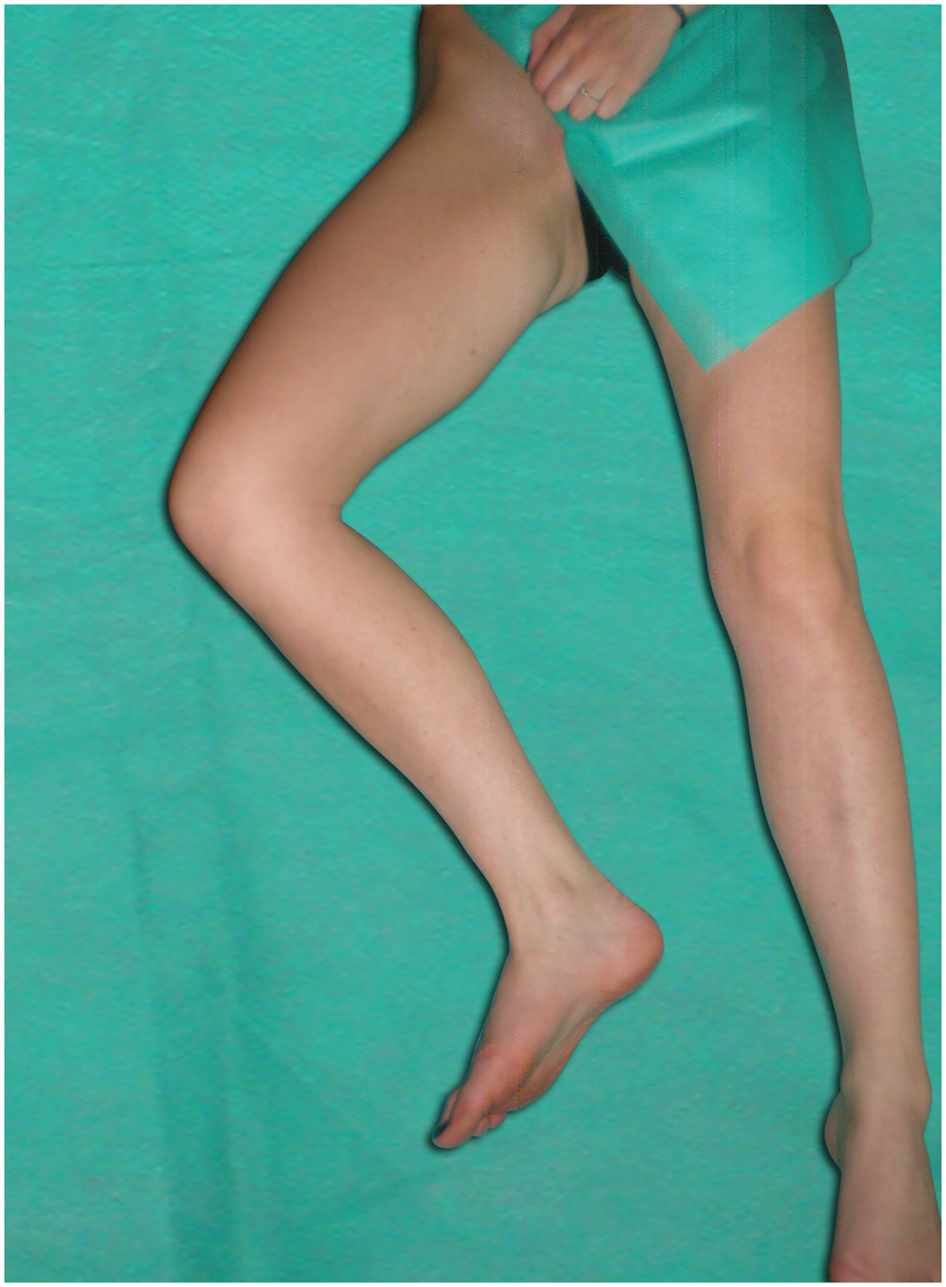
Leg position: Abduction + external rotation + 90° knee flexion/frog-leg.

The cross-sectional area (CSA) of the right femoral vein was examined at both levels in transverse plane and was measured automatically after manual tracing of the vessel on a static image in three leg positions: abduction (Fig 2), abduction + external rotation (Fig 3), abduction + external rotation + 90° knee flexion/frog-leg (Fig 4). Attention was taken to avoid any compression or displacement of the examined vein. Ultrasound measurements performed at each level in three leg positions were as follow:

Transducer position 1—measurement performed at the proximal level in abduction
Transducer position 2—measurement performed at the proximal level in abduction + external rotation
Transducer position 3—measurement performed at the proximal level in abduction + external rotation + 90° knee flexion/frog-leg
Transducer position 4—measurement performed at the distal level in abduction
Transducer position 5—measurement performed at the distal level in abduction + external rotation
Transducer position 6—measurement performed at the distal level in abduction + external rotation + 90° knee flexion/frog-leg

Study protocol can be found at: protocols.io (S1 File)

### Statistical analysis

Statistical analyses were performed using SPSS software (IBM, Chicago, IL, USA). To identify outliers we used the Grubb method provided by GraphPad software (San Diego, California, USA), available online. Distributions of the variables were tested with the Shapiro-Wilk test. After checking for normality of distribution we used repeated measure ANOVA to test a null hypothesis of no differences in mean values of CSA of the right femoral vein obtained from 3 different leg positions. We used Bonferroni correction to keep the experiment-wise error rate to a specified level alpha <0.05. To verify the hypothesis of no difference in mean CSA values obtained from two measurement levels in each leg position we used the paired-t test. We used a multivariable linear regression analysis to determine associations between CSA of the right femoral vein (dependent variable) and BMI (also weight and height separately), sex and distance from the inguinal ligament to the inguinal crease. For all statistical tests p<0.05 was considered significant.

## Results

The inguinal ligament was identified at an average distance of 22.2 mm above the inguinal crease (range limits: 14.2 mm—30.3 mm). Significant positive correlation was found between subject’s height and distance from the inguinal ligament to the inguinal crease (0.385, p<0.01), as well as between sex and distance from the inguinal ligament to the inguinal crease (0.506, p<0.01). The distance between the inguinal ligament and the inguinal crease was significantly greater in males than in females (25.3 mm, range limits: 19.6 mm—30.3 mm vs. 21.3 mm, range limits: 14.2 mm—28.1 mm).

We did not find significant outliers in CSA measurements in all leg positions and measurements locations except one significant outlier in the transducer position number 6. Subsequently, we removed this outlier from the further analysis. Distributions of data from the measurements fit Gaussian distribution.

We found significant differences in mean values of CSA obtained from the right femoral vein, which was measured at the proximal level (20 mm caudally to the inguinal ligament) in three different leg positions (F = 415.5; p<0.001). The largest mean value of CSA (114 mm^2^ ± 35 mm^2^) was found at the leg position as shown in Fig 4 and denoted as transducer position number 3, while in transducer positions 1 and 2 the mean values of CSA were significantly smaller: 69 mm^2^ ± 31 mm^2^ and 85 mm^2^ ± 31 mm^2^, respectively. Also, the differences in mean values of CSA obtained at transducer positions 1 and 2 were significant.

At the distal level (20 mm caudally to the inguinal crease) the differences in mean values of CSA of the right femoral vein in three different leg positions were significant (F = 387.9; p = 0.000). As expected, the largest CSA was found at the leg position as shown in Fig 4 (83 mm^2^ ± 37 mm^2^) and denoted as transducer position number 6, while in transducer positions 4 and 5 the mean values of the CSA were significantly smaller (51 mm^2^ ± 25 mm^2^, and 64 mm^2^ ± 30 mm^2^, respectively). The differences in mean CSA values measured in transducer positions 4 and 5 were also significant. Pair-wise comparisons of CSA values obtained in each leg position at the proximal and distal levels were significant (paired t-test).

There was a significant association of the CSA values obtained from the right femoral vein with sex and height after controlling for the effect of age, at the site of measurements 20 mm caudally to the inguinal ligament in transducer position number 1:

$$CSA\left(mm^{2}\right)=-14.90•Sex\left(p=0.015\right)+75.32•Height\left(p=0.025\right)+0.48•\;Age\left(p=0.457\right)-65.23\left(p=0.254\right)$$

Sex (male=1, female=0). (R2=0.033, F=2.29, p=0.080).

We have also found a significant association of the CSA values obtained from the right femoral vein with sex after controlling for confounding effects of height and age, at the site of measurements 20 mm caudally to the inguinal crease in transducer position number 4:

$$CSA\left(mm^{2}\right)=14.07•Sex\left(p=0.004\right)+7.81•Height\left(p=0.768\right)-0.95•Age\left(p=0.066\right)+52.34\left(p=0.252\right)$$

Sex (male=1, female=0). (R2=0.087, F=6.35, p=0.000).

There was a trend toward statistically significant differences in the CSA of the right femoral vein between males and females at the proximal measurements level. The CSA of the femoral vein in males was significantly greater than in females in all leg positions at the distal measurements level (Table 2).

**Table 2:** The cross-sectional area [mm^2^] of the right femoral vein in males and females at the proximal and distal levels in different leg positions.

		Transducer positions					
		1	2	3	4	5	6
Males n = 97	Mean	67.0	83.8	121.0	57.9	72.8	93.3
	SD	32.8	33.3	39.5	28.3	34.9	42.4
	Median	60.0	80.0	113.0	54.0	68.0	90.0
Females n = 108	Mean	71.4	86.3	109.1	44.6	56.9	75.8
	SD	28.5	29.4	30.2	19.9	23.5	28.8
	Median	72.0	83.0	109.5	41.5	54.0	74.5
	p	0.079	0.301	0.059	0.001	0.002	0.002

Transducer position 1—proximal level, abduction

Transducer position 2—proximal level, abduction+external rotation

Transducer position 3—proximal level, abduction+external rotation+90° knee flexion/frog-leg

Transducer position 4—distal level, abduction

Transducer position 5—distal level, abduction+external rotation

Transducer position 6—distal level, abduction+external rotation+90° knee flexion/frog-leg

The mean values, standard deviation, and quartile values for CSA in leg positions number 1, 3, 4, and 6 for whole group (males and females combined) are provided in Table 3.

**Table 3:** The mean values, standard deviation, and quartile values for the cross-sectional area (CSA) of the right femoral vein in leg positions 1, 3, 4, and 6 (males and females combined).

Parameter	CSA (mm^2^) Transducer Position 1 n = 205	CSA (mm^2^) Transducer Position 3 n = 205	CSA (mm^2^) Transducer Position 4 n = 205	CSA (mm^2^) Transducer Position 6 n = 204
Mean	69.3	114.7	50.9	83.4
SD	30.7	35.3	25.1	35.5
Quartile 1	45.0	90.0	31.5	58.0
Quartile 2	67.0	111.0	46.0	77.0
Quartile 3	87.0	136.0	66.0	101.0

Transducer position 1—proximal level, abduction

Transducer position 3—proximal level, abduction + external rotation + 90° knee flexion/frog-leg

Transducer position 4—distal level, abduction

Transducer position 6—distal level, abduction + external rotation + 90° knee flexion/frog-leg

The data set underlying this study is available at: https://doi.org/10.6084/m9.figshare.5255554.v1

## Discussion

Central venous cannulation via femoral vein is considered a relatively safe procedure. However, it is associated with mechanical complications, such as inadvertent arterial punctures and hematomas. Different techniques were proposed to minimize these risks by temporarily increasing the diameter of the femoral vein and therefore make it more accessible for puncture. One of the possibilities to enlarge the size of the femoral vein is to increase abdominal pressure. Among proposed methods are the Valsalva maneuver, PEEP, and manual abdominal compression. Also reversed Trendelenburg position was advised to increase the size of the vessel [9, 10]. Another approach focuses on the position of the lower limb on the side where the puncture is planned. This method seems to be more feasible, time-effective and reproducible. We undertook a study to find what the results of abduction, rotation, and knee flexion of the lower limb are in a large group of healthy volunteers and to study if there are any differences between the size of the femoral vein in men and women.

We found the cross-sectional area of the right femoral vein substantially larger in patients in the frog-leg position compared to the CSA in patients in the plain abduction leg position. The CSA was found larger independently of the site of ultrasonographic measurements in relation to the inguinal ligament. These findings may imply that in the frog-leg position the rate of successful punctures can be higher. However, this hypothesis needs to be verified in a separate study because we did not determine the rate of successful punctures nor the rate of complications. In few individual subjects, we did not observe any CSA increase with the change of the leg position, whereas in a single case the CSA actually decreased. It can justify the use of ultrasonographic guidance for further facilitating the puncture. The increase of the CSA with external leg rotation and leg flexion is most likely related to the temporary increase in intraluminal blood pressure in response to vessel’s slight compression in the inguinal triangle.

Our findings are consistent with results of other Authors, who investigated the effect of lower limb position on the femoral vein’s size and noted a significant increase of vessel’s diameter after leg abduction and rotation [11–13]. In papers by Randall et al., and Werner et al. also the influence of leg’s position on arterial overlap over the femoral vein was investigated. In both studies, a decrease of this overlap was noted after abduction and external rotation of the lower limb, which could make femoral vein easier accessible for puncture. Interesting observations come from two studies performed by Hopkins et al. and Suk et al. [14, 15], in which the Authors measured the degree of overlap in a pediatric population. In the study by Suk et al. rotation combined with abduction of the lower limb by 60° not only increased vessels’ CSA but also decreased the degree of overlap, while Hopkins et al. found that frog-leg position actually slightly increased the degree of overlap. This effect might be related to differences in study protocols: Suk et al. performed examinations on anesthetized patients, while Hopkins et al. examined non-anesthetized patients held by their parents, which might cause external compression on the inguinal region and biased measurements of the femoral vein’s size. In our study, the degree of overlap was not measured, but we decided to focus on the changes of vein’s size. We measured the CSA by delineating the circumference of the femoral vein using an electronic marker, while others used calipers to measure the vessel’s diameter [11–14]. Therefore, direct comparison of results among various studies is not straightforward. We believe our measurements are more reliable because (in contrast to arteries) veins’ walls are easily compressible and the shape of their cross-sectional area is often not perfectly circular. Calculations of the CSA based on measuring only the diameter of the vein can be biased. For instance, while the “diameter” of the vessel changes, the CSA may remain the same because of inverse changes in the size of the vein in the transverse and anteroposterior dimensions. This is why we decided to use more reliable estimates of the actual CSA based on circumferential measurements.

Another difference from other papers is that our study population was larger than others (n = 205 vs. n = 25-n = 133) and we could investigate differences in measurements between men and women, as well as the influence of height on the vein’s size.

We found a significant association of the CSA of the right femoral vein and gender after controlling for height and age. The size of the vein, however, was found different in both genders in relation to the site of CSA measurements. The median value of CSA at the proximal site, 20 mm below the inguinal ligament, is about 12 mm^2^ larger in women, whereas at the distal level, 20 mm below the inguinal crease, it is about 12.5 mm^2^ larger in men. It is not clear why this inverse relation occurs. One of the possible explanations is greater muscle mass in men, which could cause some degree of compression of the femoral vein in the upper thigh. This observation could be also related to the anatomical differences between men and women in regards to the number and location of the vein’s valves. It has been shown that females have a significantly shorter mean distance of the second distal valve to the saphenofemoral junction in the right femoral vein [16].

Also, the gender specific significant differences in distances between the inguinal ligament and the inguinal crease could contribute to the observed anatomical distinctness. This result, however, can have some clinical implications because it would be optimal to puncture the vein at the slightly more proximal site in women and slightly more caudal site in men.

As expected, the CSA of the femoral vein at the proximal site of measurements is also associated with the size of the body as determined by subject’s height. This association is not significant, however, at more caudal level. Substantial variability in the anatomical configuration of vein’s valves and sites of the great saphenous vein confluence with femoral vein can explain this lack of association [16, 17].

We have found associations of the CSA of the right femoral vein with changes of leg position, sex, and height despite substantial variability in the CSA. This variability needs to be taken into account when performing a central venous catheterization. Puncturing small veins (diameter < 7 mm) is more difficult and is related to higher complication rate [18]. In our study, 25% of veins had CSA smaller than 45 mm^2^ at the proximal level, and 31.5 mm^2^ at the more caudal level, which are related to diameters of only 5.3 mm and 4.5 mm, respectively. It is impossible to determine a priori what the size of the femoral vein is, without resorting to any imaging technique. It implies that routine use of ultrasonographic imaging prior to the puncture can facilitate the procedure and limit the complication rate [6, 19, 20].

## Conclusions

The CSA of the femoral vein is the largest in the frog-leg position in both proximal and distal levels in relation to the inguinal ligament. The CSA in women is larger closer to the inguinal ligament, while in men the CSA is larger more caudally. Substantial variability of the CSA of the femoral vein among subjects and a high proportion of relatively small vessels (diameter about 5 mm) require the use of ultrasonographic imaging when performing femoral vein puncture.

## Supporting information

S1 File Study protocol. www.protocols.io, DOI: dx.doi.org/10.17504/protocols.io.iq5cdy6.
(DOCX)
