# Supplementary material for: Cross-sectional area of the femoral vein varies with leg position and distance from the inguinal ligament
Source: PLoS One. 2017 Aug 14;12(8):e0182623. doi: 10.1371/journal.pone.0182623 (PMC5555629; doi:10.1371/journal.pone.0182623)
Supplement: S1 File — www.protocols.io, DOI: dx.doi.org/10.17504/protocols.io.iq5cdy6. (DOCX) [file pone.0182623.s001.docx]

Study protocol available on: [www.protocols.io](http://www.protocols.io), doi:https://dx.doi.org/10.17504/protocols.io.iq5cdy6

Ultrasonographic measurements of the right femoral vein cross-sectional area

Dorota Czyzewska1, Andrzej Ustymowicz2, Radoslaw Kowalewski3, Anna Zurada4, Jaroslaw Krejza5

1First Department of Radiology, Maria Sklodowska-Curie Memorial Cancer Center, Institute of Oncology, Warsaw, Poland

2Department of Radiology, Medical University of Bialystok, Bialystok, Poland

3Department of Vascular Surgery and Transplantation, Medical University of Bialystok, Bialystok, Poland

4Department of Anatomy, Faculty of Medicine University of Varmia and Mazury in Olsztyn, Olsztyn, Poland

5Institute of Innovative Medicine, Advanced Biomedical Image Laboratory, Bialystok, Poland.

Description

Exclusion criteria: lower limb varices, history of leg surgery, trauma, infection, or thromboembolic events.

Ultrasound examinations are performed in B-mode technique, equipped with a linear broadband (7.5-14-MHz) transducer. The right femoral vein and the inguinal ligament are examined in a supine position. The inguinal ligament is identified along its longitudinal axis. The measurements levels are identified as follow:

- proximal level: 20 mm caudally to the inguinal ligament identified along the femoral artery imaged with US

- distal level: 20 mm caudally to the inguinal crease visually identified and along the femoral artery imaged with US

The cross-sectional area (CSA) of the right femoral vein is examined at both levels in transverse plane and is measured automatically after manual tracing of the vessel on a static image in three leg positions: abduction, abduction + external rotation, abduction + external rotation + 90º knee flexion/frog-leg. Attention must be taken to avoid any compression or displacement of the examined vein.

DOI

dx.doi.org/10.17504/protocols.io.iq5cdy6

Contact

Dorota Czyzewska

Keywords (invisible tags)

ultrasoud, femoral vein, cross-sectional area, diameter, leg position
